# Extracellular vesicles as regulators of kidney function and disease

**DOI:** 10.1186/s40635-020-00306-2

**Published:** 2020-12-18

**Authors:** Felix Behrens, Johannes Holle, Wolfgang M. Kuebler, Szandor Simmons

**Affiliations:** 1 Institute of Physiology, Charité – Universitätsmedizin Berlin, Charitéplatz 1, 10117 Berlin, Germany; 2 Department of Pediatric Gastroenterology, Nephrology and Metabolic Diseases, Charité – Universitätsmedizin Berlin, Augustenburger Platz 1, 13353 Berlin, Germany; 3 DZHK (German Centre for Cardiovascular Research), partner site Berlin, 10117 Berlin, Germany; 4 The Keenan Research Centre for Biomedical Science at St. Michael’s, Toronto, Canada; 5 Departments of Surgery and Physiology, University of Toronto, Toronto, Canada

**Keywords:** Extracellular vesicles, Acute kidney injury, Chronic kidney disease, Sepsis, Renal replacement therapy, Dialysis

## Abstract

Extracellular vesicles (EVs) are small, lipid bilayer-delimited particles of cellular origin that recently gained increasing attention for their potential use as diagnostic biomarkers, and beyond that for their role in intercellular communication and as regulators of homeostatic and disease processes. In acute kidney injury (AKI) and chronic kidney disease (CKD), the potential use of EVs as diagnostic and prognostic markers has been evaluated in a series of clinical studies and contributions to pathophysiologic pathways have been investigated in experimental models. While EV concentrations in biofluids could not distinguish renal patients from healthy subjects or determine disease progression, specific EV subpopulations have been identified that may provide useful diagnostic and prognostic tools in AKI. Specific EV subpopulations are also associated with clinical complications in sepsis-induced AKI and in CKD. Beyond their role as biomarkers, pathophysiologic involvement of EVs has been shown in hemolytic uremic syndrome- and sepsis-induced AKI as well as in cardiovascular complications of CKD. On the other hand, some endogenously formed or therapeutically applied EVs demonstrate protective effects pointing toward their usefulness as emerging treatment strategy in kidney disease.

## Background

Acute kidney injury (AKI) is one of the major complications in critical care medicine. More than 50% of critically ill patients develop AKI, which is associated with increased in-hospital mortality [1]. AKI is defined as either

- Increase in serum creatinine (sCr) by ≥ 0.3 mg/dL (≥ 26.5 μmol/L) within 48 h, or
- Increase in sCr to ≥ 1.5 times baseline (within the last 7 days), or
- Urine volume < 0.5 mL/kg/h for 6 h [2].

sCr and urine output are also the criteria for current AKI staging according to the guidelines by Kidney Disease: Improving Global Outcomes (KDIGO; an independent non-profit foundation established by the National Kidney Foundation) from 2012, as listed in Table 1 [2].

**Table 1:** Staging of AKI [2]

Stage	sCr	Urine output
1	▪ 1.5–1.9 times baseline, or ▪ ≥ 0.3 mg/dL (≥ 26.5 mmol/L) increase	< 0.5 mL/kg/h for 6–12 h
2	2.0–2.9 times baseline	< 0.5 mL/kg/h for ≥ 12 h
3	▪ ≥ 3.0 times baseline, or ▪ increase to ≥ 4.0 mg/dL (≥ 353.6 mmol/L), or ▪ initiation of renal replacement therapy, or ▪ in patients < 18 years, decrease in eGFR to < 35 mL/min per 1.73 m^2^	▪ < 0.3 mL/kg/h for ≥ 24 h, or ▪ anuria for ≥ 12 h

eGFR estimated glomerular filtration rate. Modified from “KDIGO Clinical Practice Guideline for Acute Kidney Injury”

**Fig. 1:**
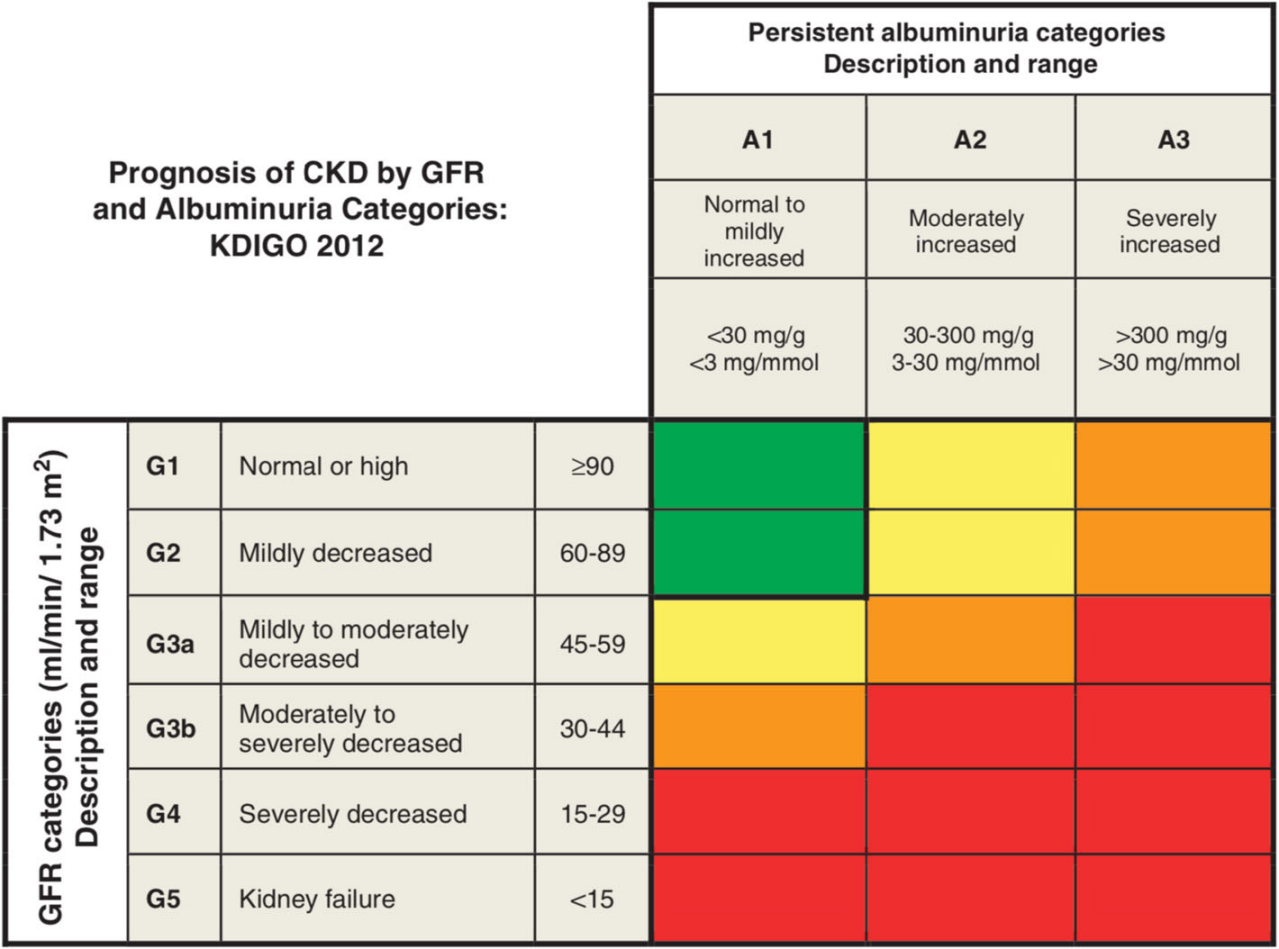
Staging of CKD. Green: low risk (if no other markers of kidney disease, no CKD); yellow: moderately increased risk; orange: high risk; red: very high risk. From “KDIGO 2012 Clinical Practice Guideline for the Evaluation and Management of Chronic Kidney Disease” with kind permission by Kidney International Supplements [12]

Until recently, the classification of acute renal failure did not follow a uniform standard until AKI as a term was established. The definition of acute renal failure used to describe the disease by its etiology and anatomical origin, classifying it in prerenal, intrinsic renal, and postrenal kidney failure, with the intrinsic renal causes subdivided in vascular diseases, glomerulonephritis, interstitial nephritis, and ischemic or nephrotoxic tubular necrosis [3]. In contrast, the definition of AKI only relies on sCr and urine volume as parameters of kidney function, allowing the allocation to subgroups with graded outcomes [4, 5]. However, as treatment or removal of the underlying condition is the first and ideal line of therapy in AKI [6], the present definition and classification of AKI are currently under debate for their neglect to account for the actual cause kidney injury [7]. Most cases of AKI collectively share morphological characteristics like tubular damage [8], while tubulointerstitial pathology is also common in chronic kidney disease (CKD) [9]. Such links between AKI and CKD are not confined to associations at the pathological level, such as subclinical AKI episodes leading to increased tubulointerstitial fibrosis [10], but are also frequent in the clinical context: AKI is a major risk factor for the development of CKD, and conversely, diagnosed CKD increases the incidence and aggravates the outcome of AKI [4, 10, 11]. Hence, AKI and CKD are increasingly considered not to be separate diseases but rather closely connected syndromes [4, 10]. Currently, CKD is defined as “abnormalities of kidney structure or function, present for > 3 months, with implications for health” [12], diagnosed by fulfilling at least one of the following criteria for more than 3 months:

- Glomerular filtration rate (GFR) < 60 mL/min/1.73 m^2^
- Albuminuria (albumin excretion rate ≥ 30 mg/24 h; albumin-to-creatinine ratio ≥ 30 mg/g [≥ 3 mg/mmol])
- Urine sediment abnormalities
- Electrolyte and other abnormalities due to tubular disorders
- Abnormalities detected by histology
- Structural abnormalities detected by imaging
- History of kidney transplantation

and staged by albuminuria and GFR, as shown in Fig. 1 [12]. The global prevalence of CKD is currently estimated at 8-16% with diabetes and hypertension as the dominant causes in all developed and many developing countries [13]. With a growing population affected by type 2 diabetes [14], hypertension [15], and metabolic syndrome [16], the prevalence of CKD is predicted to increase to even higher levels, thus awareness for CKD is increasingly required especially as systemic pharmacological interventions often need to be adjusted according to impaired kidney function. Current guidelines recommend the use of sCr or, in case of CKD, estimated GFR (eGFR) calculated on the base of sCr for the diagnosis of kidney damage [2, 12]. However, it is important to keep in mind that sCr and kidney function do not show a linear correlation and accordingly, small increases in sCr can already reflect marked functional declines in the initial phase of kidney diseases [17], complicating early diagnosis of AKI and especially CKD.

An improved awareness and workup for early diagnosis is not the only aspect of impaired kidney function that demands further attention. At present, our pathophysiological concepts cannot fully explain central aspects of kidney disease in detail, e.g., the molecular and cellular basis of tubulointerstitial fibrosis as characteristic feature of CKD [18] or how kidney disease induces the well-described molecular mechanisms leading to the development of vascular calcification as common complication of CKD [19]. Recently, extracellular vesicles (EVs) have gained attention for their potential as diagnostic markers, as mediators of intercellular communication and dissemination of homeostatic and disease signals, and as modulators of target cell transcriptomics, proteomics, lipidomics, and function in healthy and disease processes [20, 21]. In this review, we will highlight the emerging potential of EVs as diagnostic biomarkers, contributors to pathophysiological mechanisms of disease progression, and as therapeutic targets in kidney disease.

## Biogenesis, characterization, and distribution of extracellular vesicles

**Fig. 2:**
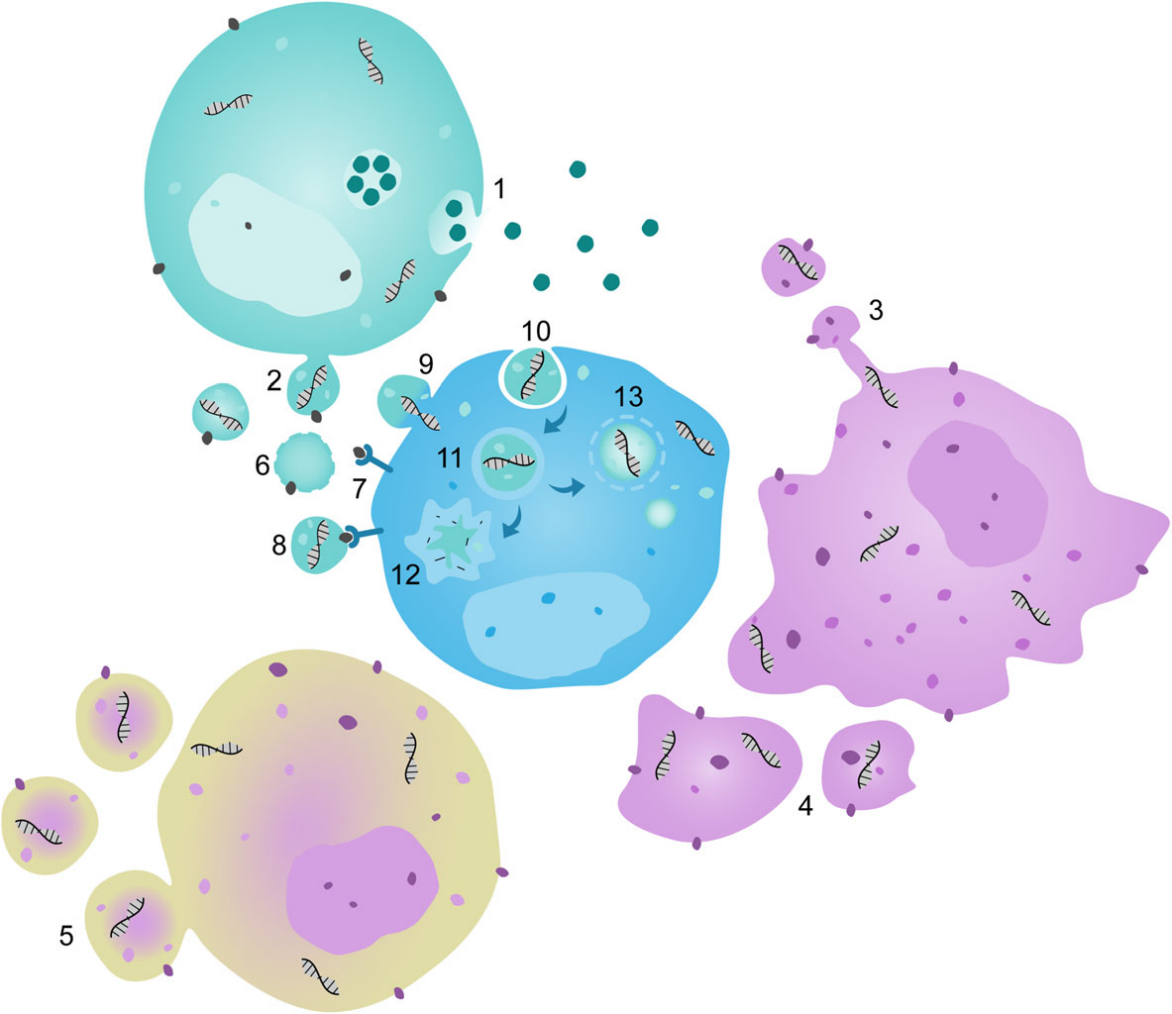
Biogenesis of EVs and mechanisms of signal transduction. Exosomes are released from cells into the extracellular space by fusion of endosomes (multivesicular bodies) with the plasma membrane of the cell (1). In contrast, microvesicles are formed directly at the plasma membrane and bud from its lipid bilayer (2). In apoptotic cells, membrane protrusions are formed and release vesicles from their top (3), and apoptotic bodies of heterogenic size and morphology are generated as the cell dissolves (4). In tumor cells large EVs termed oncosomes can bleb of the plasma membrane into the extracellular space (5). There are several ways how EVs can transmit information to target cells: (i) they can undergo lysis in the extracellular space (6), releasing their cargo and membrane components, which can then bind to receptors on cell surfaces (7); (ii) surface molecules of intact EVs can stimulate receptors on target cells (8); and (iii) EVs can be incorporated by cells, and release their content through membrane fusion (9), or undergo endocytosis (10) being transferred into endosomes (11) with either lysosomal degradation (12), or escape from the endosome delivering the EV’s cargo into the target cell’s cytoplasm (13). Modified from “Extracellular Vesicles: Unique Intercellular Delivery Vehicles” [21]

EVs are lipid bilayer-delimited particles of cellular origin, which in contrast to cells are unable to replicate [22]. They can be classified by their biogenesis in exosomes, microvesicles (ectosomes, microparticles), and larger EVs (apoptotic bodies, oncosomes), as shown in Fig. 2. The smallest class of EVs are exosomes, commonly considered to be below 150 nm, which are formed within endosomes (multivesicular bodies), and which are released into the extracellular space by fusion of the endosome with the plasma membrane. In contrast, microvesicles with sizes ranging from 100 to 1000 nm [23] are formed upon plasma membrane budding, using the lipid bilayer of its cell of origin and, therefore, sharing (part of) its characteristic membrane proteins [21, 24]. In cancer cells, even larger EVs, termed oncosomes, have been described, which are formed by membrane blebbing, ranging from 1 to 10 μm in size. Apoptotic bodies are heterogenous in their size and can reach similar dimension as oncosomes, but are in general formed by dying cells [21]. As especially exosomes and smaller microvesicles reveal a considerable overlap in size [25], recent guidelines recommend to rather use the terms small and large EVs in samples of unclear composition [21]. EVs have been detected in virtually all biofluids, including blood, urine, cerebrospinal fluid, and bronchoalveolar lavage fluid [20]. Importantly, EVs are not—as initially believed—mere “cellular dust” but actively transport specific proteins, lipids, RNAs, most relevantly mRNAs, miRNAs and small interfering RNAs [26], cell organelles, and in apoptotic bodies also higher levels of genomic DNA [27]. As such, EVs act as important intercellular shuttles of information, which can be transmitted to target cells in several ways (displayed in Fig. 2): (i) by extracellular lysis of the vesicle and release of its contents, which may then act as ligands to stimulate receptors on the target cell; (ii) by binding of EV surface molecules to receptors on the target cell; or (iii) by cellular uptake of the vesicle by either membrane fusion, releasing the EV’s cargo into the cytoplasm, or endocytosis and subsequent lysosomal degradation of or escape from the endosome in order to deliver the cargo to the cytoplasm of the target cell [21]. The small size and physicochemical heterogeneity of EVs necessitates specialized analytical methods for adequate EV detection, quantification, characterization of membrane composition, and lineage tracing [22]. Flow cytometry is one of the most commonly used methods for EV detection that allows simultaneous lineage tracing by staining with specific antibodies for selected surface molecules [28], which led to the identification of frequent populations of EVs in the blood, with respective lineage-specific surface markers (Table 2).

**Table 2:** Cellular lineages and characteristic surface markers of frequent circulating and urinary EVs

Compartment	EV population	Parent cell	EV surface markers
Blood	EC-EVs	Endothelial cells	CD31, CD34, CD62E, CD105, CD106, CD142, CD144, CD146
	leu-EVs ▪ B-EVs ▪ Mo-EVs ▪ PMN-EVs - Nφ-EVs ▪ T-EVs	Leukocytes ▪ B lymphocytes ▪ monocytes ▪ granulocytes - neutrophils ▪ T lymphocytes	CD11a, CD18, CD45 ▪ CD19, CD20 ▪ CD14, CD16, CD33, CD64, CD142 ▪ CD11b, CD15 - CD16, CD66b ▪ CD3, CD4, CD8
	plt-EVs	Platelets	CD31, CD41a, CD42a, CD42b, CD61, CD62P
	RBC-EVs	Erythrocytes	CD35, CD235a, CD235b
Urine	Pod-EVs	Podocytes	KIRREL (NEPH1), Nephrin, Podocalyxin, Podocin (NEPH2)
	TEC-EVs	Tubular epithelial cells	Aquaporin 1 (AQP1), CD10, CD13, Uromodulin (Tamm Horsfall Glykoprotein)
	CDEC-EVs	Collecting duct epithelial cells	Aquaporin 2 (AQP2)

Although flow cytometry allows detailed phenotyping and quantification of frequencies of specific EVs in biofluids, flow cytometric analysis has disadvantages in exact quantification of EV size and concentration in these fluids. Therefore, nanoparticle tracking analysis that relies on Brownian molecular motion for the quantification of particles remains the preferred method for accurate and detailed enumeration of size distribution and concentration of EVs in the respective biofluids [22]. Finally, in order to confirm the membrane-delimited character of the particles detected by flow cytometry or nanoparticle tracking analysis and to clearly differentiate them from non-vesicular aggregates or complexes, electron microscopy is indispensable [29]. Since there is no single method allowing for enumeration, characterization, and lineage tracing of EVs at the same time, current consensus initiatives aim to standardize the study of EVs [22]. The present use of different analytical approaches for EVs frequently restricts direct comparison between different studies.

In kidney disease, EVs from blood and urine appear as potential biomarkers or modulators of disease processes. Notably, the cellular origin of urinary EVs (uEVs) is very different from plasma EVs [30], which usually have diverse cellular descent (Table 2), e.g., from endothelial or circulating blood cells [31], or are derived from cellular lineages of perfused organs and their parenchyma, e.g. the liver [32]. Under physiological conditions, these circulating EVs cannot pass through the glomerular membrane and are confined to the plasma space [33]. In contrast, uEVs typically derive by more than 99% from cells in the urinary tract, mostly podocytes, tubular cells, and the epithelium of the collecting duct (Table 2) [34]. As such, uEVs represent an independent functional and diagnostic tool relative to EVs from other compartments [30]. Apart from the assessment of the biomarker suitability in kidney disease, EVs were described in different biofluids in renal replacement therapy (RRT), e.g., peritoneal dialysis (PD) effluent, which pronounces the manifold opportunities EVs imply also in clinical monitoring of treatment regimes.

## Extracellular vesicles in acute kidney injury

Pathophysiological alterations associated with AKI are expected to start before the disease is detectable by increased sCr or reduced urine excretion. Therefore, early identification of patients at high risk for AKI is of central importance to warrant effective intervention strategies. In addition, the identification of new biomarkers may promote our understanding of the molecular basis of the disease or even guide the way to new therapeutic strategies. Hence, a growing body of work is focusing on reliable risk predictors, which recently included an increasing interest on EVs as diagnostic tool for AKI. Here, we highlight first the implications of EVs in AKI, and afterwards focus on sepsis and hemolytic-uremic syndrome (HUS) as two important causes of AKI, in which the function of EVs were extensively studied (as summarized in Table 3).

**Table 3:** Changes in EV characteristics upon different forms of kidney disease

Condition	EV populations and cargo^a^	Function^b^
AKI	EC-EVs ↑, plt-EVs ↑ [35] uEVs: AQP1 ↓, fetuin-A ↑, ATF3 ↑ [36–38]	
▪ Sepsis	▪ leu-EVs ↑ (B-EVs, Mo-EVs, PMN-EVs, T-EVs) [42–48] protein: A2MG ↑, β2-integrin ↑, EPCR ↑, PD-L2 ↑, PS ↑, TF ↑, thrombomodulin ↑ [46, 47, 52, 53, 55, 57, 62] miRNA: miR-21-5p ↓, miR-193a-5p ↓ [64] mRNA: MPO ↑, FOXM1 ↑, SELS ↑, GLRX2 ↑, PRDX3 ↑, SOD2 ↑ [65] Sepsis-AKI: PS^+^ EVs ↑, PS^+^ plt-EVs ↑, PS^+^/CD13^+^ EVs ↑, β2-integrin ↑ [57]	▪ leu-EVs: bacterial growth ↓ [43] A2MG^+^ PMN-EVs: bacterial load ↓, hypothermia ↓, leukocyte count ↓ (peritoneal exudate, lung tissue), mortality ↓ [63] thrombin ↑, factor X ↑ [42, 47] heart, lung: eNOS ↑, SOD ↑, iNOS ↑, COX-2 ↑, NF-κB ↑ [66] liver: eNOS ↓, SOD2 ↓, COX-2 ↓, I-κBα phosphorylation ↓ [66]
▪ HUS	▪ leu-EVs ↑, plt-EVs ↑, RBC-EVs ↑, C3^+^ and C9^+^ EVs (plt, Mo, Nφ) ↑ [70–72] protein: C3 ↑, C9 ↑, TF ↑ [72, 75]	▪ plt-EVs, Mo-EVs, Nφ-EVs, RBC-EVs: Stx transport and uptake into renal ECs, podocytes, tubular epithelium [77]
CKD	EC-EVs ↑, PS^+^ (EC-) EVs ↑ [81, 82, 84–91] protein: GRP ↓, fetuin-A ↓ [97] miRNA: miR-223 ↑ [81] uEVs: CD2-associated protein mRNA ↓ [98]	thrombin ↑, osteocalcin ↑ (VSMCs, EPCs, fibroblasts), VSMC osteochondrogenic differentiation and inflammation ↑, VSMC calcification ↑, angiogenesis ↓, EC apoptosis ↑, endothelium-dependent relaxation ↓, endothelial cGMP and NO ↓ [81, 82, 87, 91, 97] miR-223^+^ EVs: VSMC calcification ↑, angiogenesis ↓, EC apoptosis ↑ [81]

ATF3 activating transcription factor 3, EPCR endothelial protein C receptor, FOXM1 forkhead box protein M1, GLRX2 glutaredoxin 2, GRP gla-rich protein, MPO myeloperoxidase, PRDX3 peroxiredoxin 3, SELS selenoprotein S, VSMC vascular smooth muscle cell

^a^If not declared differently, EVs were purified from patients’ blood

^b^Note that EV functions are commonly evaluated in bulk preparations and not in the specifically altered subpopulations. If specific subpopulations are given, those EVs were produced in vitro

In order to address the potential utility of EVs as predictive markers, it is critical to do so in prospective rather than cross-sectional or retrospective studies and to compare EV characteristics between patients who develop AKI and subjects who do not. To our knowledge, this criterion has only been fulfilled in a single pilot study by Sullo et al. [35], in which children undergoing cardiac surgery with cardiopulmonary bypass were assessed. Here, no significant differences in concentration and size distribution of plasma EVs were detected between patients who developed AKI within the first week after surgery as compared to those without AKI, although fractions of platelet (plt)- and endothelial cell (EC)-derived EVs were increased in AKI patients [35].

Other groups analyzed uEVs to distinguish patients with high AKI risk from those with lower complication probability. Insights provided by preclinical animal studies and small patient cohorts suggest the usefulness of aquaporin-1, fetuin-A, and activating transcription factor 3 concentrations in urinary exosomes as predictive biomarkers for AKI [36–38]. In animal models, that induced renal failure by either ischemia/reperfusion injury (IRI) or cisplatin treatment, urinary exosomal aquaporin-1 was already decreased to about half of the baseline level within in the first 6 h after injury [36], while first fetuin-A increases were detectable after 24 h [37]. Activating transcription factor 3 increased in the first 2 h after ischemia and reperfusion, while first increases were detectable 24 h after cisplatin treatment without reaching equally high levels as in IRI [38]. These results indicate the potential expediency of urinary exosomal activating transcription factor 3 and especially aquaporin-1 as very early predictors for AKI in routine clinical practice. Aquaporin-1 was not only found to be decreased in uEVs in AKI, but—in line with uEVs’ derivation from the renal parenchyma—was also downregulated in the total kidney in IRI, suggesting its potential pathomechanistic involvement [36]. None of these markers have so far been tested prospectively in humans, but cross-sectional pilot studies demonstrate promising potential for the use of uEVs in the early diagnosis of AKI. Specifically, urinary exosomal fetuin-A was shown to be elevated in a few AKI patients as compared to healthy donors [37]. Interestingly, exosomal activating transcription factor 3 was already detected at timepoints when sCr increases were still subtle and may thus present a particularly early biomarker of AKI [38].

### Extracellular vesicles in sepsis

Sepsis is a life-threatening condition characterized by a dysregulated response to infection causing not only organ dysfunction but also high morbidity and mortality (17%) [39, 40]. Sepsis is the most frequent cause of AKI in critically ill patients, accounting for about half of AKI cases in intensive care unit patients [41]. In the strive for the development of new diagnostic and therapeutic tools, EVs have recently emerged as mediators that can directly induce and/or accelerate sepsis. In line with a dysregulated inflammatory status in sepsis, several studies detected elevated concentrations of EVs of leukocyte origin (leu-EVs) in the blood of septic patients as compared to healthy donors [42–48], including (i) EVs derived from granulocytes (PMNs) [42, 43, 45–47]; (ii) monocyte (Mo)-derived EVs [45–48]; (iii) T-lymphocyte-derived EVs [46, 47]; and (iv) EVs derived from B cells [47]. As sepsis is frequently associated with endothelial dysfunction [49] and a prothrombotic state [50], a growing body of work has also screened for EC- and plt-EVs and found blood EC-EV levels to be increased in septic patients [46, 47, 51–53], although this change was not evident in all studies [54]. In addition, increased levels of plt-EVs have been reported [42, 47, 55–57], yet again not consistently [46, 51]. No matter whether these incongruencies are attributable to heterogeneities in the underlying cause of sepsis [58, 59], the host response, or the time course and severity of the disease, they question the current usability of EV-subpopulations as diagnostic and likely, also prognostic marker for the disease. Whether improved patient stratification, e.g., according to pathogens and primary route of infection, may potentially increase the usefulness of EVs as reliable indicators of sepsis remains to be shown. In this respect, a potential effect of the underlying infection and/or the pharmacological therapy of sepsis should be taken into consideration as additional potential modulator of EV titers in biofluids. In accordance with this notion, the antibiotic sulfisoxazole has been shown to inhibit the release of EVs by binding to endothelin receptor A [60]. Hence, it is worth to consider that pharmacological interventions may significantly reduce the diagnostic value of EVs; an aspect that should be taken into account for future study designs. Yet, even if EC- and plt-EVs do not appear to present valid diagnostic markers to detect patients at risk for sepsis, several associations with clinical outcome are noteworthy and may hint to their prognostic relevance as well as to underlying pathomechanisms. For instance, plt-EV concentrations were elevated in septic patients that would die within the next 48 h compared to those who would survive this period [52]. In another study, EC-EV levels correlated positively with development of disseminated intravascular coagulation [61], supporting results that link procoagulant molecules to EVs in sepsis patients, including phosphatidylserine (PS) [46, 47, 55, 57] and tissue factor [47, 53]. When comparing EVs from patients with sepsis and renal failure with EVs from septic patients without renal failure, Tokes-Fuzesi et al. identified increased total PS^+^ EVs, PS^+^ plt-EVs, and PS^+^/CD13^+^ myeloid EVs on the day of admission in the blood of patients who already developed AKI by that time [57]. One may therefore speculate if kidney failure aggravates the prothrombotic state in sepsis by an increase in PS^+^ EVs or if patients with established organ failure just represent an advanced stage of the disease with an equally increased risk of thrombotic events. In line with these findings on procoagulant EVs, sepsis EVs have been shown to have direct prothrombotic effects by enhancing thrombin [42, 47] and factor X [47] generation. However, the anticoagulant proteins thrombomodulin and endothelial protein C receptor are also elevated on EVs from septic patients compared to healthy donors [53], suggesting that EVs play a pleiotropic role in coagulation abnormalities in sepsis. It will be of interest in further studies to determine whether the distribution of pro- and anticoagulant molecules and their influence on hemostasis can be linked to specific EV-lineage subpopulations. Other proteins have been associated with EVs in septic patients including β2-integrin, PD-L2 (programmed cell death 1 ligand 2), and alpha-2-macroglobulin (A2MG). As such, β2-integrin and PD-L2 levels are not only elevated in EVs of septic patients compared to healthy donors, but increased β2-integrin concentrations also correlated with hypotension and reduced kidney function [62]. A2MG^+^ EVs are elevated in community acquired pneumonia sepsis compared to fecal peritonitis sepsis and healthy donors [46]. Notably, A2MG^+^/PS^+^ EV concentrations were increased at the onset of sepsis in survivors as compared to non-survivors and healthy controls [63], indicating a potential adaptive/protective EV-mediated response. In line with this view, A2MG-enriched EVs produced by human PMNs not only decreased bacterial load in blood and peritoneal exudate, alleviated hypothermia and decreased leukocyte counts in peritoneal exudate and lung tissue, but also significantly reduced overall mortality in a preclinical model of fecal peritonitis-induced murine sepsis following cecal ligation and puncture [63]. These results suggest a potential role of A2MG^+^ EVs as host defense mechanism and offer promising potential for the use as future intervention strategy. Another interesting, possibly therapeutic, aspect is the antibacterial effect of leu-EVs: EVs derived from PMNs of healthy donors that were stimulated with opsonized *Staphylococcus aureus* bacteria inhibited bacterial growth [43], implying that EVs play a role in the activation of innate immunity.

Apart from protein cargo of EVs and their functional importance, several studies focused on the characterization of EV RNA-content in sepsis. Reithmair et al. reported 15 down- and 25 upregulated miRNAs in septic EVs compared to those from healthy individuals [64]. Decreased exosomal miR-27b-3p, miR-21-5p, and miR-193a-5p were associated with disease severity, while miR-21-5p and miR-193a-5p were significantly decreased in EVs from patients’ blood compared to EVs from healthy donors. In addition, a decrease in exosomal miR-30a-5p and miR-125b-5p appears to be associated with mortality [64], but still has to be validated as biomarker in a subsequent prospective cohort. Various mRNAs related to oxidative stress (myeloperoxidase, forkhead box protein M1, selenoprotein S, glutaredoxin 2, peroxiredoxin 3, superoxide dismutase 2 (SOD2)) were increased in EVs already at the day of diagnosis, indicating that EVs may contribute to an antioxidative host response in sepsis [65]. In line with such an adaptive role for EVs, Mastronardi et al. administered EVs from human septic patients to wild-type mice and found expression of endothelial nitric oxide synthase (eNOS) and extracellular SOD to be elevated in heart and lung of sepsis EV-treated mice when compared to treatment with EVs from healthy controls [66]. Yet, in contrast to the presumed protective effects of eNOS and SOD, inducible nitric oxide synthase (iNOS), cyclooxygenase-2 (COX-2) and NF-κB (nuclear factor kappa-light-chain-enhancer of activated B cells) were also elevated in their expression in heart and lung of sepsis EV-treated mice, while in the liver eNOS and SOD2 were decreased, but so were COX-2 levels and IκBα (nuclear factor of kappa light polypeptide gene enhancer in B-cells inhibitor, alpha) phosphorylation [66]. Thus, in heart, lung, and liver, both protective, antioxidative, and anti-inflammatory and detrimental, pro-oxidative, and pro-inflammatory effects seem to be induced in parallel, indicating that overall EVs from septic patients’ blood have pleiotropic effects on oxidative and inflammatory metabolism. In the kidney, no changes in NO and superoxide anion production could be detected [66].

In conclusion, a considerable body of research has recently advanced our understanding of the role of EVs not only as biomarkers of sepsis, but rather as functional effectors or modulators in a variety of pathomechanisms including disseminated intravascular coagulation and oxidative stress. These insights may help to provide for a better clinical assessment of sepsis in terms of prognosis, and establish the role of EVs as at least in part protective effectors with regenerative potential as adaptive mechanism in sepsis. In addition, these studies highlight the pharmacological potential of EVs or their cargo for future therapeutic approaches, e.g., to induce an adequate antibacterial host response. The relationship between EVs and the pathomechanisms of kidney injury and failure in sepsis, however, has presently not been extensively addressed and requires future preclinical and clinical research to delineate a potential regulatory role of EVs.

### Extracellular vesicles in hemolytic uremic syndrome

A special case of AKI that has gained public attention after an outbreak of enterohemorrhagic *Escherichia coli* (EHEC/STEC) in Germany in 2011 [67] is HUS. Shiga toxin-induced hemolytic-uremic syndrome (STEC-HUS) is induced by intoxications with EHEC and represents the vast majority of HUS cases [68]. Shiga toxin (Stx) is commonly described as the main mediator of the disease and delivers its toxicity by inhibition of the protein synthesis in target cells [69]. Although not the most frequent cause of AKI [6], substantial efforts have been made to advance diagnostics and the pathophysiological understanding of the disease, including the analysis of EVs. As such, Ge et al. [70] reported elevated concentrations of plt-EVs and leu-EVs in the plasma of patients with STEC-HUS compared to healthy donors. These findings were confirmed by Ståhl et al. who found complement factor (C3 and C9) positive plt-EVs, Mo-EVs, and neutrophil (Nφ)-derived EVs to be elevated in plasma in the acute phase of STEC-HUS [71]. Concentrations of red blood cell (RBC)-derived EVs have also been found increased in both pediatric and adult STEC-HUS patients compared to healthy controls [72]. In pediatric STEC-HUS patients, these RBC-EVs also bore an increased amount of complement factors (C3 and C9) shortly after diagnosis [72]. It is thus tempting to speculate that EVs take part in the initiation of pathologic processes in STEC-HUS, including a prothrombotic state and hemolysis [73]. In earlier works, plt-EVs and Mo-EVs induced by Stx and plasma EVs of acute phase STEC-HUS patients were also found to carry high levels of tissue factor, a classic initiator of hemostasis [74], compared to EVs from vehicle-treated cells and healthy donor EVs, respectively [75]. Again, these findings may indicate a pathogenic contribution of EVs to the procoagulant state in STEC-HUS. Similarly, EVs in patients with nephrotic syndrome have been identified as procoagulant, yet with PS identified as procoagulant mediator rather than tissue factor [76]. However, if in STEC-HUS increased coagulation is mediated through TF or like in nephrotic syndrome, the effect is at least partially mediated by EV-derived PS remains elusive. Over and above that, EVs may also contribute in additional ways to renal failure in STEC-HUS since plt-EVs, Mo-EVs, Nφ-EVs, and RBC-EVs have been identified as carriers for the cytotoxic Stx and mediate its uptake (i) into the renal endothelium, as demonstrated in a human renal biopsy sample, and (ii) apparently also into podocytes and the tubular epithelium, as shown in EHEC infected BALB/c mice [77]. Given that extensive endothelial and tubular damage in the kidney is a consequence of STEC-HUS, EV-mediated Stx dissemination and uptake may provide for a novel (and potentially targetable) explanation for progressive tubular damage in STEC-HUS, although these findings will have to be tested in larger cohorts before a definite pathomechanism may be defined.

As tubular damage is the common denominator and manifestation of kidney injury from various insults, these findings may suggest an emerging role of EVs not only as a promising future diagnostic tool in early risk assessment of AKI but also as important players in disease pathogenesis and interorgan crosstalk. Whether and if so, how EVs control tubular damage and which biological active molecules they may shuttle into target cells are hence important questions that remain to be resolved.

## Extracellular vesicles in chronic kidney disease

Similar to AKI, diagnosis of CKD at an early stage of the disease is a challenge, as clinical symptoms tend to arise at later stages and since the most commonly used markers to estimate GFR, e.g., sCr, are influenced by nutrition, physical activity, and muscle mass [78]. Thus, there is an unmet clinical need for diagnostic and predictive biomarkers, especially considering that early diagnosis is critical for prompt initiation of, e.g., anti-hypertensive treatment to protract the progression of CKD to end-stage renal disease (ESRD) [79, 80]. Recent efforts focusing on the evaluation of EVs in the blood of CKD patients revealed elevated total EV levels in patients not yet eligible for RRT and in patients upon treatment by hemodialysis (HD), hemodiafiltration (HDF), or PD compared to healthy donors [81–83]. In these patient groups, a considerable body of work has characterized the cellular origin of blood EVs, their membrane composition, and cargo (summarized in Table 3). EC-EV concentrations were found to be elevated in patients not yet eligible for RRT and in patients treated with HD or PD when compared to healthy subjects [81, 82, 84–91]. Yet, differences between those three patient groups could only be detected in a pediatric cohort, in which HD and PD patients’ blood contained higher EC-EV levels than samples from patients not yet eligible for RRT [88]. Notably, both elevated CD144^+^ and CD146^+^ EC-EV levels were correlated with increased pulse wave velocity, a classic marker of arterial stiffening, while no correlation was found between CD146^+^ EVs with hemoglobin levels in the blood or GFR [88], which may point toward a regulatory influence of EC-EV cargo on vascular calcification and/or remodeling. Several studies also reported elevations in plt-EVs in CKD patients compared to healthy controls [82, 83, 86, 87, 92]. However, there seems to be a considerable variability in circulating plt-EVs, as one study only detected increased plt-EV levels for patients not yet eligible for RRT and not for HD patients [86], while another analysis found significant differences merely between healthy controls and HD patients after their dialysis sessions and not before [93]. Other studies did not detect any significant changes of plt-EVs in CKD patients compared to healthy donors [81, 94] or in one case also not for EC-EVs [94]. These inconsistencies preclude a meaningful prognostic or diagnostic interpretation of EV levels in CKD. Both preanalytical conditions, such as sample storage time, and the analytical procedures themselves, which currently include different methods of EV enrichment and quantification, would need to be standardized in order to potentially generate less variable results that would be key for the potential use as biomarker. In addition, thorough time course analyses would be required as a potential source for the observed differences. In a limited number of studies, leu- [86]/Nφ-EVs [93] and RBC-EVs [87] were demonstrated to be elevated in HD patients, while no changes in leu-EV concentrations could be found in patients not yet eligible for RRT [86]; PD patients were not assessed in these evaluations. As these analyses were only performed in small patient cohorts, the evaluation of leu- and RBC-EVs should be continued in larger groups of patients in order to investigate their possible association with inflammatory state and anemia, both commonly described in CKD patients [95, 96].

Concerning protein cargo, EVs from ESRD patients not yet eligible for RRT showed decreased levels of gla-rich protein and fetuin-A [97]. This aspect is interesting since in AKI uEVs bore more fetuin-A than uEVs of healthy subjects [37]. Hence, a ratio of fetuin-A carrying EVs in blood versus urine may potentially emerge as tool to predict development and progression of kidney injury. uEVs in CKD were only evaluated in a single study by Lv et al. who reported decreased levels of CD2-associated protein mRNAs in uEVs of CKD patients compared to healthy controls [98]. CD2-associated protein is a cytoskeletal protein and deficiencies in it are associated with increased risk of glomerular disease [99]. This may act as a potential confounder in the study by Lv and colleagues as patients with glomerular disease were included into the study [98]. With respect to EV membrane composition, PS^+^ EC-EVs were found elevated in HD patients in one cohort compared to healthy donors [85], while total PS^+^ EVs were increased in patients not yet eligible for RRT and HD patients in another study [86]. In vitro experiments also showed increased PS^+^ EV release from ECs after stimulation with the uremic toxins p-cresol and indoxyl sulfate [86]. Considering the procoagulant potential of PS^+^ EVs in AKI [76], PS^+^ EVs may present relevant mediators of hemostasis in CKD as the disease strongly associates with thrombotic events [100]. In support of this notion, a correlation of elevated plt-EV levels and thrombotic events was found in uremic patients [92] and CKD patients’ EVs were shown to enhance thrombin formation [82].

**Fig. 3:**
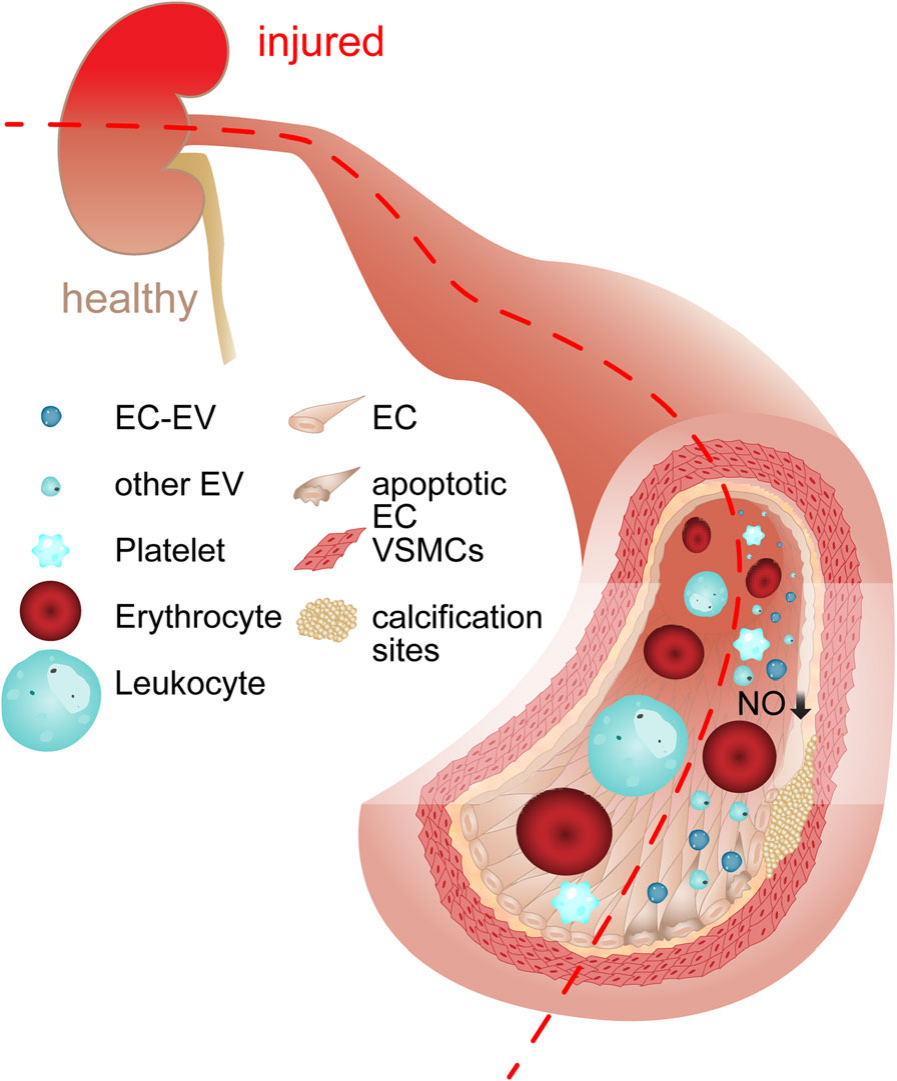
Vascular effects of circulating EVs from patients with kidney injury. Blood EVs from patients with CKD convey pathologic effects on the vessel wall compared to EVs from healthy donors in vitro. These include increased endothelial apoptosis, reduced angiogenesis (not displayed), vascular calcification by VSMC osteochondrogenic differentiation and reduced endothelial NO production as a marker for endothelial dysfunction. The concentration of EC-EVs in patient samples revealed a positive correlation with these effects, pointing towards a potential pathologic effect of EC-EVs in the promotion of vascular complications in CKD

In parallel, EVs may also be associated with pathomechanisms driving other cardiovascular complications in CKD, in as much as blood EC-EV levels correlated with pulse wave velocity [87, 88], common carotid artery augmentation index and a loss of flow-mediated dilation as markers of vascular stiffening [87]. Consistent with an association of EVs with vascular complications of CKD, EC-EV concentrations were also increased in CKD patients with vascular calcification compared to CKD patients without vascular calcification and were inversely associated with a decrease in endothelial progenitor cells (EPCs) [91]. Moreover, CKD patients’ blood EVs from the same cohort induced osteocalcin expression in EPCs of healthy donors, VSMCs, and fibroblasts [91], pointing to a potential pathogenic role for EVs in vascular calcification what goes in line with the previously mentioned findings that showed decreased levels of fetuin-A, which is known to inhibit vascular calcification, in blood EVs from CKD patients [19]. Such calcifying effects of CKD EVs were confirmed by Viegas et al. [97] who demonstrated the induction of VSMC osteochondrogenic differentiation and inflammation by patients’ EVs, while Cavallari et al. [81] identified VSMC calcification elicited by HD patient-derived EVs. The latter experiments also revealed inhibition of angiogenesis and increased endothelial cell apoptosis in response to EVs, further consolidating the functional link to vascular pathologies associated with CKD. These effects could be linked to miR-223 as potential mediator, as this specific miRNA was elevated in patients not yet eligible for RRT, PD, and HD patients, and inhibition of miR-223 alleviated the observed pathologic effects in vitro [81]. Further strengthening the pathologic connection to cardiovascular disease, EVs could also be linked to endothelial dysfunction in CKD, as treating rat aortic rings with EVs from CKD patients reduced endothelium-dependent relaxation and cGMP and NO production compared to treatment with EVs from healthy donors while these markers of endothelial dysfunction were correlated with EC-EV levels in the blood of the patients, from which EVs were obtained [87]. The pathologic vascular effects of EVs from CKD patients are summarized in Fig. 3. A recent study in patients with coronary artery disease and CKD provides interesting insights into the potential disseminating or aggravating effects of EVs on vascular disease in CKD patients. Here, coronary artery disease was associated with elevated blood plt- and EC-EVs in both CKD patients and subjects without renal disease in comparison to healthy controls, while no significant difference in EV concentrations was found between coronary artery disease patients with or without CKD [94]. These findings raise the question whether differences in EV characteristics in CKD patients compared to healthy controls may rather reflect the vascular disease that accompanies CKD in advanced stages [13] than indicating decreasing kidney function directly. In cardiovascular disease, EVs are well-established critical mediators of pathophysiological processes, e.g., impaired vasodilation in acute coronary syndrome [101], and the findings listed above may suggest a similar role for cardiovascular complications in CKD. As such, it will be important for future research to clearly differentiate the role of EVs in kidney disease per se versus associated cardiovascular comorbidities in CKD.

Regarding the diagnostic and predictive use of EVs in CKD, EC- and especially plt-EVs have so far proven difficult to compare in cross-sectional studies, presumably again due to different EV isolation/enrichment and quantification techniques and/or different timepoints studied. However, longitudinal studies show promise for the use of EVs to identify high-risk patients in that elevated EC-EV levels were associated with increased total mortality [85, 102] and cardiovascular events (myocardial infarction, cerebrovascular accident, transient ischemic attack) [85]/cardiovascular death [102] in studies following HD patients for more than 5 years.

## Extracellular vesicles in renal replacement therapy

ESRD and AKI patients in advanced stages require RRT to treat the life-threatening complications of kidney failure, including volume overload, acidosis, electrolyte abnormalities, and uremia [103]. While the risk for CKD patients to develop ESRD requiring RRT within 5 years of diagnosis is below 20% even at G4 stage [104], the majority (> 70%) of AKI patients on intensive care units requires RRT [105]. When considering the reported differences in EV characteristics in patients treated with different forms of RRT, including HD, HDF, and PD, compared to pre-RRT patients and healthy controls, it is important to evaluate not only the influence of the stage of the underlying disease on EV characteristics but also to bear in mind to analyze the potential effects of RRT techniques on EV status (Table 4). Extracorporeal approaches of RRT include HD, hemofiltration, HDF as a combination of principles used in HD and hemofiltration, isolated ultrafiltration, plasmapheresis, and plasma-/hemoperfusion [106], with intermittent HD and continuous venovenous hemofiltration (CVVH) being the most commonly used in intensive care unit patients [107] and intermittent HD in ambulatory settings [108]. Interestingly, intact EVs are not efficiently removed from the blood in CVVH treatment, as no EVs were detectable in the ultrafiltrate and as plasma EV concentrations were unaltered before and after filtration [109], indicating that current membranes of RRT devices are largely impermeable for EVs. This notion is supported by an earlier study that examined the level of circulating miRNAs in HD-treated patients. Similar to EVs, miRNA concentrations did not differ between pre-dialysis and post-dialysis blood samples and only minimal traces of miRNA could be detected in the dialysate and ultrafiltrate [110]. This observation is consistent with a preferred shuttling of miRNAs by larger structures such as EVs, and supports the notion that the latter cannot be efficiently removed by HD. In CVVH-treated patients with sepsis-induced AKI blood-derived EV, characteristics were compared pre- to post-filter, which revealed an increase in CD31^+^/CD41^−^ EVs behind the filtration device [109]. Although CD31 is generally considered as a classic endothelial marker [111], it is also expressed—yet at lower levels—on granulocytes, monocytes, and platelets [112]. Since the elevated EVs were CD41-negative and thus not platelet-derived, the authors concluded that the detected CD31^+^/CD41^−^ EVs may reflect an increase in leu-EVs as a result of CVVH treatment [109]. However, circulating endothelial cells, which have recently emerged as biomarkers for both renal and cardiovascular disease, should probably also be considered as alternative source [113].

**Table 4:** Influence of RRT on EV properties in CKD.

Condition	EV populations and cargo^a^	Function^b^
CVVH	Ultrafiltrate: no EVs [109] pre- vs. post-filter: total EVs →, CD31^+^/CD41^-^ EVs ↑ (sepsis-AKI) [109]	
HD	Ultrafiltrate/dialysate: only traces of miRNA (hence no EVs) [110] pre- vs. post-HD: total plasma miRNA → [110] low flux: EC-EVs and Mo-EVs ↑ after HD compared to high flux [116]	miR-223^+^ EVs: VSMC calcification ↑, angiogenesis ↓, EC apoptosis ↑ [81]
HDF	EC-EVs ↓ compared to HD [81, 90, 117] miR-223 ↓ compared to HD [81]	VSMC calcification ↓, angiogenesis ↑, EC apoptosis ↓ compared to HD [81]
PD	PD effluent: mesothelial EVs detected [119, 120]	

^a^If not declared differently, EVs were purified from patients’ blood

^b^Note that EV functions are commonly evaluated in bulk preparations and not in the specifically altered subpopulations

A series of studies have addressed changes in EV quality and quantity before and after one dialysis session and reported EC-EVs [114, 115] and also total EVs, plt-, and leu-EVs [115] to be decreased after HD treatment. Reduced EC-EVs levels were associated with higher brachial laminar shear stress in patients with arteriovenous fistulas as vascular access for HD treatment [114]. In contrast, Nφ- and plt-EVs were elevated after dialysis [93] and a variety of EVs including plt-, Mo-, and EC-EVs were increased at 1 h into HD treatment [116]. Hence, there is no discernible overall trend whether extracorporeal RRT induces EV generation or not and according to current findings, the filter unit of the dialysis device does not effectively eliminate EVs.

A considerable body of work has also compared EV characteristics between different RRT methods. Low-flux HD-treated patients bore a higher increase in EC- and Mo-EV levels during their dialysis sessions compared to high-flux treated subjects [116]. Conversely, patients treated with the recently developed online HDF had significantly lower blood EC-EV levels compared to patients treated with regular HD [81, 90, 117]. Cavallari et al. also showed reduced miR-223 concentrations within plasma EVs of online HDF patients and demonstrated reconstitution of angiogenesis and a decrease in endothelial apoptosis and VSMC calcification compared to plasma EVs from HD-treated patients [81]. This protective cardiovascular effect uncovers a therapeutically interesting side-aspect of online HDF and as such may position this technique as a preferred RRT method. Novel RRT evolutions include the use of HDF with endogenous reinfusion and mid-dilution, an extension of HDF aimed to enhance the clearance of middle and larger molecular weight substances. As both modalities decreased EC-EV levels compared to HD [89, 90], it will be of specific interest to see whether these procedures concomitantly also reduce cardiovascular outcome and overall mortality. So far, it remains unclear whether EC-EVs are solely responsible for cardiovascular morbidity, as analyses on both EV cargo and function were performed with bulk EV preparations of different cellular origins. Preparative flow-cytometric isolation and functional testing of defined EV populations has previously been described [118], but has not received appreciation in many studies yet. In addition to an uncertain cellular origin of potentially pathogenic EVs, it is also yet to be specified whether alterations in EV characteristics of RRT-treated patients as compared to healthy donors are an expression of ESRD or rather the applied invasive treatment regime. A “double hit” hypothesis appears reasonable in this regard: with the onset of CKD-specific alterations in EV composition begin as the “first hit” and advance with the progression of the disease. The initiation of extracorporeal RRT reflects the “second hit,” which goes in line with clinically visible worsening of the cardiovascular phenotype, to which the previously mentioned EVs with harmful effects on the vasculature might contribute. However, this is a hypothesis that will have to be tested in extensive comparisons of not only the EV phenotype but also their functional relevance in different stages, cardiovascular phenotypes, and treatment modes of CKD, which would be ideal to probe in longitudinal studies.

In PD, EVs could be detected in the PD effluent [119–121] and a fraction of those showed characteristics of a mesothelial origin [119, 120]. In addition, peptide profiles in EVs obtained from PD effluent differed between newly enrolled subjects in comparison to longer treated patients [121]. This gives rise to the notion that PD efflux EVs might function as markers for PD efficiency and peritoneal membrane status. Peritoneal fibrosis in prolonged PD treatment, which is associated with decreased ultrafiltration capacity, is commonly only diagnosed upon the onset of clinical symptoms when the pathological process is already advanced [122]. Potential functions of intraperitoneal EVs as biomarkers or even as active mediators in peritoneal fibrosis would pave the way for new diagnostic or therapeutic avenues, yet the usefulness and validity of such an approach remains to be evaluated.

## Extracellular vesicles in the therapy of kidney failure

At present, treatment options for kidney failure remain limited. In AKI, efforts aim to apply specific, largely pharmacological therapies to address the underlying cause [123]. However, most patients only receive supportive therapy and the number of effective targeted treatments is restricted [124, 125]. In CKD, the situation is equally bleak. Even in current guidelines, therapeutic options are largely considered in the context of “Prevention of CKD progression” [12], reflecting the present view that CKD is an irreversibly advancing disease, and that its treatment is currently limited to protracting progression. This apparent gap in treatment options stresses the need for novel approaches and strategies for the treatment of kidney failure.

### Administration of progenitor cell-derived EVs

Over the past years, a considerable body of work has started to investigate the potential of different stem cell-based therapies in kidney disease [126, 127], primarily focusing on either autologous or allogenic transplantation of mesenchymal stem cells (MSC) [127]. The mechanisms by which stem cells alleviate kidney damage are not completely understood, yet it has become evident over the past decade that stem cells do not engraft in sufficient numbers to restore organ function, but rather act via the release of paracrine mediators. This recognition has fueled the interest in EVs as candidate mediators of MSCs’ curative effects [128]. Considering that MSC functionality, specifically metabolic activity, proliferation, and paracrine communication, is impaired in the presence of the uremic toxins p-cresol and indoxyl sulfate [129], EVs administered with MSC transplantation or released immediately thereafter rather than MSCs themselves may in fact constitute the main or part of the functional component responsible for the documented renoprotective effects. Evidence for EVs’ therapeutic potential has been shown in different animal models of AKI, in which EVs harvested in vitro from MSCs were administered and induced morphologic and functional recovery, e.g., by enhancing tubular repair and angiogenesis, reducing renal fibrosis, and modulating immune cell infiltration [128, 130, 131]. Renal damage was likewise attenuated by MSC-EVs in an extracorporeal model of cold ischemia mimicking explanted organs for transplantation [132]. Collino et al. produced EVs derived from either wild-type MSCs or from MSC following prior Drosha-knockdown [131], a common technique to deplete cellular miRNAs [133]. Cells with Drosha-knockdown produced EVs in similar quantity and membrane composition compared to wild-type MSC-EVs. They were taken up into tubular epithelial cells in the same manner as wild-type MSC-EVs but were depleted of miRNAs. As a result, they failed to induce the same regenerative effect as EVs from wild-type MSCs [131], indicating that miRNAs transported by MSC-EVs regulate kidney recovery. This notion was further confirmed at the transcriptional level in that RNA-depleted EVs were unable to cause a similar decrease in transcripts for lipocalin 2 and fibrinogen, both markers for tubular damage, in kidney tissue as compared to EVs with normal miRNA composition [131]. In addition to MSC-derived EVs, EVs derived from endothelial colony-forming cells, EPCs, and tubular epithelial cells were tested. EVs from endothelial colony-forming cells decreased tubular cell death in IRI mice and attenuated endothelial apoptosis in vitro [134], while EPC-EVs in IRI rats enhanced tubular cell proliferation and reduced apoptosis and leukocyte infiltration [135]. In the same study, some IRI animals were housed for 6 months as a preclinical model of CKD. Strikingly, in the EPC-EV-treated group of rats, a significant reduction in capillary rarefication, glomerulosclerosis, and tubulointerstitial fibrosis could be observed when compared to the control group. EVs bearing the proangiogenic miR-126 and miR-296 were identified to contribute to this renoprotective effect, as depletion of EV RNA by treatment with RNase or specific miR-antagomirs for miR-126 and miR-296 or Dicer-knockdown in the EV-generating cells caused a loss of the protective effects [135]. EVs from rat tubular epithelial cells also led to faster recovery from IRI in rats and decreased morphologic abnormalities [136]. In addition, EV treatment reduced ischemia-induced oxidative stress and post-IRI fibrosis. Interestingly, in this series of experiments, the authors could also conclusively illustrate effective ischemic preconditioning via an EV-mediated effect in that all protective effects were increased when EVs were harvested from cells exposed to hypoxia [136]. These experiments were subsequently also validated by the same group for EVs from human tubular epithelial cells [137]. In conclusion, EVs have recently emerged as central mediators of the therapeutic potential of stem cell therapy in kidney injury, raising the possibility of a cell-free cell therapy with clear advantages in terms of storage, safety, and production.

It should, however, be considered that most of these promising results were generated in animal models of AKI, with EV treatment administered at a definite time either before or after conditional AKI induction. This allows for in-depth investigation of mechanisms of action in experimental models of CKD on the one hand, and for the translation of findings from preclinical studies into the patient on the other hand. However, the therapeutic implementation of targeted EV therapy for patients with AKI will likely be more complex, considering that the determination of a specific timepoint, at which the intervention is to be initiated, is difficult due to the lack of early predictive markers. In addition, patient-specific (i.e., autologous) cell isolation, EV production, and enrichment are time-consuming processes [138] and are not feasible in scenarios of sudden kidney failure that require immediate treatment. Thus, novel therapeutic approaches may build upon the promising renoprotective effects of progenitor cell-derived EVs but implement proven beneficial components into artificial nanocarriers with similar characteristics as EVs to generate a readily available, of-the-shelf product for the effective treatment of kidney injury.

### Inhibition and removal of disease-associated EVs

With advancing knowledge on EV subpopulations, one may speculate that editing potentially pathological EVs in the blood, e.g., by removing them or inhibiting them with a blocking molecule, may provide for an alternative approach. A few years ago, a method for the removal of EVs from the blood was suggested that can be easily integrated into standard HD or CVVH devices and is thus relatively easy to implement in RRT patients. In this affinity plasmapheresis platform EVs smaller than 200 nm can pass through hollow fibers, in which molecules bind specific pre-selected subgroups of EVs with high affinity and thus remove them from the circulating blood stream [139]. Despite the apparent advantages of selective removal of specific EV subpopulations, several limitations of this approach need to be considered: (i) a size restriction for efficient clearance of EVs to those with a diameter of less than 200 nm may not target the EVs with disease-promoting potential [139]; (ii) modifying the EV composition comprises risks, as deletion of homeostatic EVs may lead to systemic consequences; and (iii) as an invasive technology that requires either arteriovenous fistulas or central venous catheters, it raises the risk of infections and limited patient acceptance. Thus, the potential benefits of affinity plasmapheresis need to be carefully weighed against these potential adverse effects. An alternative therapeutic strategy is the pharmacological inactivation of specific pathological EVs in vivo. Proof of principle for the feasibility of such an approach has been demonstrated by two studies, in which therapeutic antibodies were shown to opsonize tumor-derived EVs from breast cancer and b cell lymphoma cells [140, 141]. Even though only the binding of the antibodies to EVs has been proven and not their removal, and although the identification of practical target EV subpopulations will require further research, there seems to be enormous potential in the use of antibody-mediated EV editing as a personalized medicine approach for diseases that are yet not properly treatable.

## Discussion

In different forms of kidney disease, EVs including a variety of subpopulations have been identified in blood and urine and are associated with clinical outcome. While in sepsis, as the most frequent cause of AKI, EVs may predict disseminated intravascular coagulation and mortality, in CKD they associate with cardiovascular complications as a frequent cause of co-morbidity and mortality [142]. Notably, EVs from CKD patients directly induce vascular dysfunction and especially calcification, and should hence not only be considered as biomarkers, but rather as mediators of the underlying pathologic processes. In the treatment of ESRD patients, these calcifying effects of EVs from CKD patients also revealed the advantages of new HDF methods as compared to HD. Pathophysiological contributions of EVs were also demonstrated in STEC-HUS, where EVs promote a procoagulant state and take part in the distribution of Stx, and in sepsis, where EVs mediate hemostasis. As the latter role is likely not restricted to sepsis, EVs may play an important role in pathologic coagulation in various diseases but also modulate hemostasis under physiological conditions. On the other hand, EVs do not only bear detrimental effects, as shown, e.g., by A2MG^+^ EVs, which improved the outcome in septic mice and were also detected in blood from human septic patients, hinting toward a potential adaptive host defense mechanism that may potentially be exploited for treatment. A therapeutic approach that has already been tested in animal models is the infusion of EVs from stem or progenitor cells in kidney diseases, especially AKI. While preclinical results are promising, the translation to humans remains difficult, as harvesting of autologous stem and progenitor cells, cell expansion, and isolation/purification of autologous EVs still requires too much time for effective treatment in acute scenarios such as AKI, in particular when the diagnosis cannot be made at an early stage of the disease due to subtle or non-specific clinical symptoms.

As a roadmap for the future clinical use of EVs in kidney disease, several objectives will have to be resolved before EV diagnostics and therapy can be translated from bench to bedside and become useful tools on a day-to-day basis. Firstly, in EV diagnostics, standardization in both pre-analytical and analytical steps will have to be enhanced since current studies tend to lack comparability. In addition, the present cross-sectional studies will have to be complemented by longitudinal studies that test the robustness of diagnostic markers of renal insufficiency, treatment efficacy, and related comorbidities identified by the comparison of patients at different disease stages and healthy individuals. Ideally, these studies would not only follow diseased patients through their progression but also identify the predictive value of certain EV populations in larger cohort studies. In the therapy of kidney disease, EVs derived from progenitor cells have proven their value in preliminary studies, but difficulties in the production of such EVs prevent a broader application of this approach so far. The identification of their beneficial components might help to overcome current obstacles, since it may allow for the selective loading of EVs with these components or for the design of artificial nanocarriers, which share the beneficial characteristics but may in the end become a ready-to-use product in contrast to the time-consuming harvesting of EVs from autologous stem cells. Independent of the success of these concepts, the evaluation of pharmacodynamics and pharmacokinetics of any treatment that involves the administration of exogenous EVs to a patient is essential before this kind of EV therapy can advance from the experimental phase to a clinical strategy. Over and above that, editing of circulating EVs by either inhibition or removal of pathologic EVs is a strategy that may complement the use of therapeutic EVs in the scope of personalized medicine in intensive care. While the technical requirements appear to be in reach, the selection of the detrimental populations of EVs remains challenging. So far, most functional analyses of EVs in kidney disease were performed using bulk preparations, and different populations were only evaluated in diagnostics. Therefore, it will be essential to identify distinct subpopulations of EVs that bear adverse effects in order to be able to achieve the goal of modifying endogenous EV populations as a therapeutic approach. Taking into account these promising prospects not only in diagnostics but also in the therapy of kidney disease, it seems that so far, we are only seeing the tip of the iceberg of the many functions and possibilities of EVs.

## Conclusion

In the past decade, EVs have become identified as mediators of intercellular communication, and as biomarkers and potential propagators or modulators of pathologic processes in renal disease. Accordingly, an increasing body of work elucidates links between fundamental aspects of EV biology, specifically conditions of release, trafficking, targeting abilities, uptake routes, and bio-distribution profiles of EVs, with the pathophysiology of kidney disease. Consequently, EVs have emerged as both diagnostic biomarkers of and pathophysiologic contributors to various kidney diseases, including acute conditions like HUS and sepsis-induced AKI, but also CKD. The differentiation of the heterogenous population of EVs into subtypes for functional analysis, and further characterization of their cargo as well as physical and functional properties is challenging. While recent technical advances have identified specific subpopulations of EVs that can differentiate patients with kidney disease from healthy individuals, their prognostic reliability remains elusive, even if first studies in this regard show promising results. Furthermore, the functional relevance of EVs as mediators of disease processes but also as potential protective mechanism is complex. In order to promote the translation of the rapidly advancing knowledge regarding the role of EVs in kidney disease into clinically successful therapies, a comprehensive understanding of the underlying biology of EV cargo uptake and processing appears to be fundamental. In conjunction with the recent advances, these new insights encourage specialists in renal and cardiovascular biology, signaling, proteomics, lipidomics, and nanotechnology to join forces with clinicians in order to define, probe, and optimize the use of EVs as novel cell-free therapeutic strategy for widespread diseases like CKD.

## Data Availability

Not applicable.

## Abbreviations

A2MG: Alpha-2-macroglobulin
AKI: Acute kidney injury
AQP1: Aquaporin-1
ATF3: Activating transcription factor 3
B-EVs: B cell/B lymphocyte-derived extracellular vesicles
CKD: Chronic kidney disease
COX-2: Cyclooxygenase-2
CVVH: Continuous venovenous hemofiltration
EC: Endothelial cell
EHEC: Enterohemorrhagic *E. coli* (see also STEC)
eNOS: Endothelial nitric oxide synthase
EPC: Endothelial progenitor cell
EPCR: Endothelial protein C receptor
ESRD: End-stage renal disease
EV: Extracellular vesicle
FOXM1: forkhead box protein M1
(e)GFR: (Estimated) glomerular filtration rate
GLRX2: Glutaredoxin 2
GRP: Gla-rich protein
HD: Hemodialysis
HDF: Hemodiafiltration
HUS: Hemolytic-uremic syndrome
IκBα: Nuclear factor of kappa light polypeptide gene enhancer in B-cells inhibitor, α
iNOS: Inducible nitric oxide synthase
IRI: Ischemia/reperfusion injury
KDIGO: Kidney Disease: Improving Global Outcomes
leu: Leucocyte
Mo: Monocyte
MPO: Myeloperoxidase
MSC: Mesenchymal stem cell
Nφ: Neutrophil granulocyte
NF-κB: Nuclear factor kappa-light-chain-enhancer of activated B-cells
PD: Peritoneal dialysis
PD-L2: Programmed cell death 1 ligand 2
plt: Platelet
PMN: Granulocyte (polymorphonuclear neutrophil)
PRDX3: Peroxiredoxin 3
PS: Phosphatidylserine
RBC: Red blood cell
RRT: Renal replacement therapy
sCr: Serum creatinine
SELS: Selenoprotein S
SOD(2): Superoxide dismutase (2)
STEC: Shigatoxigenic *E. coli* (see also EHEC)
STEC-HUS: Shiga toxin-induced hemolytic-uremic syndrome
Stx: Shiga toxin
T-EVs: T cell/T lymphocyte-derived extracellular vesicles
TF: Tissue factor
uEV: Urinary extracellular vesicle
VSMC: Vascular smooth muscle cell
